# Invasive *Haemophilus influenzae* Serotype e and f Disease, England and Wales

**DOI:** 10.3201/eid1805.111738

**Published:** 2012-05

**Authors:** Shamez N. Ladhani, Sarah Collins, Anna Vickers, David J. Litt, Carina Crawford, Mary E. Ramsay, Mary P.E. Slack

**Affiliations:** Health Protection Agency, London, UK (S.N. Ladhani, S. Collins, A. Vickers, D.J. Litt, C. Crawford, M.E. Ramsay, M.P.E. Slack);; St. George’s University of London, London (S.N. Ladhani)

**Keywords:** Haemophilus influenzae, Hif, Hie, epidemiology, risk factors, MLST, outcome, serotypes, England, Wales, bacteria, respiratory tract diseases, *Suggested citation for this article*: Ladhani SN, Collins S, Vickers A, Litt DJ, Crawford D, Ramsay ME, et al. Invasive *Haemophilus influenzae* serotype e and f disease, England and Wales. Emerg Infect Dis [serial on the internet]. 2012 May [*date cited*]. http://dx.doi.org/10.3201/eid1805.111738

## Abstract

Incidence of serotype e was 3-fold lower than serotype f, but it caused more severe clinical disease.

## Medscape CME Activity

Medscape, LLC is pleased to provide online continuing medical education (CME) for this journal article, allowing clinicians the opportunity to earn CME credit.

This activity has been planned and implemented in accordance with the Essential Areas and policies of the Accreditation Council for Continuing Medical Education through the joint sponsorship of Medscape, LLC and Emerging Infectious Diseases. Medscape, LLC is accredited by the ACCME to provide continuing medical education for physicians.

Medscape, LLC designates this Journal-based CME activity for a maximum of 1 *AMA PRA Category 1 Credit(s)*^TM^. Physicians should claim only the credit commensurate with the extent of their participation in the activity.

All other clinicians completing this activity will be issued a certificate of participation. To participate in this journal CME activity: (1) review the learning objectives and author disclosures; (2) study the education content; (3) take the post-test with a 70% minimum passing score and complete the evaluation at www.medscape.org/journal/eid; (4) view/print certificate.

**Release date: April 16, 2012; Expiration date: April 16, 2013**

## Learning Objectives

Upon completion of this activity, participants will be able to:

- Analyze the epidemiology of Hie and Hif
- Distinguish the risk for mortality associated with invasive cases of Hie and Hif
- Evaluate invasive infection with Hie and Hif among children
- Evaluate invasive infection with Hie and Hif among older adults.

## CME Editor

**Shannon O’Connor,** Technical Writer/Editor, *Emerging Infectious Diseases*. *Disclosure: Shannon O’Connor has disclosed no relevant financial relationships.*

## CME Author

**Charles P. Vega, MD,** Health Sciences Clinical Professor; Residency Director, Department of Family Medicine, University of California, Irvine. *Disclosure: Charles P. Vega, MD, has disclosed no relevant financial relationships.*

## Authors

*Disclosures:* ***Shamez N. Ladhani, MRCPCH, PhD,*** *has disclosed the following relevant financial relationships: served as an advisor or consultant for GSK, Pfizer, SPMSD, Baxter; served as a speaker or a member of a speakers bureau for GSK, Pfizer, SPMSD, Baxter (all on behalf of St. Georges University of London, but has not received any personal remuneration for these activities).* ***Sarah Collins; Anna Vickers, MD; Carina Crawford; and Mary E. Ramsay, FFPHM,*** *have disclosed no relevant financial relationships.* ***David J. Litt*** *has disclosed the following relevant financial relationships: received grants for clinical research from GSK.* ***Mary P.E. Slack, FRCPATH,*** *has disclosed the following relevant financial relationships: served as a speaker or a member of a speakers’ bureau for GSK, Pfizer; received grants for clinical research from GSK, Pfizer; received support for travel to scientific meetings from GSK, Pfizer, Merck.*

## References

[1] Peltola H. Worldwide *Haemophilus influenzae* type b disease at the beginning of the 21st century: global analysis of the disease burden 25 years after the use of the polysaccharide vaccine and a decade after the advent of conjugates. Clin Microbiol Rev. 2000;13:302–17. 10.1128/CMR.13.2.302-317.2000 10756001 PMC100154

[2] Heath PT, Booy R, Azzopardi HJ, Slack MP, Bowen-Morris J, Griffiths H, Antibody concentration and clinical protection after Hib conjugate vaccination in the United Kingdom. JAMA. 2000;284:2334–40. 10.1001/jama.284.18.2334 11066183

[3] Ladhani S, Slack MP, Heys M, White J, Ramsay ME. Fall in *Haemophilus influenzae* serotype b (Hib) disease following implementation of a booster campaign. Arch Dis Child. 2008;93:665–9. 10.1136/adc.2007.126888 17942585

[4] Takala AK, Eskola J, Leinonen M, Kayhty H, Nissinen A, Pekkanen E, Reduction of oropharyngeal carriage of *Haemophilus influenzae* type b (Hib) in children immunized with an Hib conjugate vaccine. J Infect Dis. 1991;164:982–6. 10.1093/infdis/164.5.982 1940479

[5] Tsang R. Capsule switching and capsule replacement in vaccine-preventable bacterial diseases. Lancet Infect Dis. 2007;7:569–70. 10.1016/S1473-3099(07)70191-3 17714669

[6] Lowther SA, Shinoda N, Juni BA, Theodore MJ, Wang X, Jawahir SL, *Haemophilus influenzae* type b infection, vaccination, and H. influenzae carriage in children in Minnesota, 2008–2009. Epidemiol Infect. 2012;140:566–74. 10.1017/S0950268811000793 21676359

[7] Ladhani S, Slack MP, Heath PT, von Gottberg A, Chandra M, Ramsay ME. European Union Invasive Bacterial Infection Surveillance participants. Invasive *Haemophilus influenzae* disease, Europe, 1996–2006. Emerg Infect Dis. 2010;16:455–63. 20202421 10.3201/eid1603.090290 PMC3322004

[8] Rubach MP, Bender JM, Mottice S, Hanson K, Weng HY, Korgenski K, Increasing incidence of invasive *Haemophilus influenzae* disease in adults, Utah, USA. Emerg Infect Dis. 2011;17:1645–50. 10.3201/eid1709.101991 21888789 PMC3322072

[9] Shuel M, Hoang L, Law DK, Tsang R. Invasive *Haemophilus influenzae* in British Columbia: non-Hib and non-typeable strains causing disease in children and adults. Int J Infect Dis. 2011;15:e167–73. 10.1016/j.ijid.2010.10.005 21134777

[10] Resman F, Ristovski M, Ahl J, Forsgren A, Gilsdorf JR, Jasir A, Invasive disease caused by *Haemophilus influenzae* in Sweden 1997–2009; evidence of increasing incidence and clinical burden of non–type b strains. Clin Microbiol Infect. 2011;17:1638–45. 10.1111/j.1469-0691.2010.03417.x 21054663

[11] Kalies H, Siedler A, Grondahl B, Grote V, Milde-Busch A. von KR. Invasive *Haemophilus influenzae* infections in Germany: impact of non-type b serotypes in the post-vaccine era. BMC Infect Dis. 2009;9:45. 10.1186/1471-2334-9-45 19379490 PMC2678273

[12] Giufrè M, Cardines R, Caporali MG, Accogli M, D’Ancona F, Cerquetti M. Ten years of Hib vaccination in Italy: prevalence of non-encapsulated *Haemophilus influenzae* among invasive isolates and the possible impact on antibiotic resistance. Vaccine. 2011;29:3857–62. 10.1016/j.vaccine.2011.03.059 21459175

[13] Urwin G, Krohn JA, Deaver-Robinson K, Wenger JD, Farley MM. Invasive disease due to *Haemophilus influenzae* serotype f: clinical and epidemiologic characteristics in the H. influenzae serotype b vaccine era. The *Haemophilus influenzae* Study Group. Clin Infect Dis. 1996;22:1069–76. 10.1093/clinids/22.6.1069 8783712

[14] Resman F, Svensj T, Nal C, Cronqvist J, Brorson HK, Odenholt I, Necrotizing myositis and septic shock caused by *Haemophilus influenzae* type f in a previously healthy man diagnosed with an IgG3 and a mannose-binding lectin deficiency. Scand J Infect Dis. 2011;43:972–6. 10.3109/00365548.2011.589079 21728743

[15] Adam HJ, Richardson SE, Jamieson FB, Rawte P, Low DE, Fisman DN. Changing epidemiology of invasive *Haemophilus influenzae* in Ontario, Canada: evidence for herd effects and strain replacement due to Hib vaccination. Vaccine. 2010;28:4073–8. 10.1016/j.vaccine.2010.03.075 20398617

[16] MacNeil JR, Cohn AC, Farley M, Mair R, Baumbach J, Bennett N, Current epidemiology and trends in invasive *Haemophilus influenzae* disease—United States, 1989–2008. Clin Infect Dis. 2011;53:1230–6. 10.1093/cid/cir735 22080119

[17] Laboratory reports of *Haemophilus influenzae* by age group and serotype, England and Wales, fourth quarter, 2010 (and 2009). Health Protection Reports. 2011;5:6–7.

[18] Ladhani S, Slack MP, Heath PT, Ramsay ME. Changes in ascertainment of Hib and its influence on the estimation of disease incidence in the United Kingdom. Epidemiol Infect. 2007;135:861–7. 10.1017/S0950268806007382 17092395 PMC2870625

[19] Heath PT, Booy R, Griffiths H, Clutterbuck E, Azzopardi HJ, Slack MP, Clinical and immunological risk factors associated with *Haemophilus influenzae* type b conjugate vaccine failure in childhood. Clin Infect Dis. 2000;31:973–80. 10.1086/318132 11049779

[20] Slack MPE. *Haemophilus*. In: Borriello SP, Murray PR, Funke G, editors. Topley and Wilson’s microbiology and microbial infections, vol. 2. London: Hodder Arnold Ltd.; 1995. p. 1692–718.

[21] Hobson RP, Williams A, Rawal K, Pennington TH, Forbes KJ. Incidence and spread of *Haemophilus influenzae* on an Antarctic base determined using the polymerase chain reaction. Epidemiol Infect. 1995;114:93–103. 10.1017/S0950268800051943 7867747 PMC2271340

[22] van Ketel RJ. de WB, van AL. Detection of *Haemophilus influenzae* in cerebrospinal fluids by polymerase chain reaction DNA amplification. J Med Microbiol. 1990;33:271–6. 10.1099/00222615-33-4-271 2258914

[23] Falla TJ, Crook DW, Brophy LN, Maskell D, Kroll JS, Moxon ER. PCR for capsular typing of *Haemophilus influenzae.* J Clin Microbiol. 1994;32:2382–6. 7814470 10.1128/jcm.32.10.2382-2386.1994 PMC264070

[24] Meats E, Feil EJ, Stringer S, Cody AJ, Goldstein R, Kroll JS, Characterization of encapsulated and noncapsulated *Haemophilus influenzae* and determination of phylogenetic relationships by multilocus sequence typing. J Clin Microbiol. 2003;41:1623–36. 10.1128/JCM.41.4.1623-1636.2003 12682154 PMC153921

[25] Litt DJ, Neal SE, Fry NK. Changes in genetic diversity of the *Bordetella pertussis* population in the United Kingdom between 1920 and 2006 reflect vaccination coverage and emergence of a single dominant clonal type. J Clin Microbiol. 2009;47:680–8. 10.1128/JCM.01838-08 19158267 PMC2650949

[26] Campos J, Hernando M, Roman F, Perez-Vazquez M, Aracil B, Oteo J, Analysis of invasive *Haemophilus influenzae* infections after extensive vaccination against H. influenzae type b. J Clin Microbiol. 2004;42:524–9. 10.1128/JCM.42.2.524-529.2004 14766811 PMC344522

[27] Campos J, Roman F, Perez-Vazquez M, Oteo J, Aracil B, Cercenado E. Infections due to *Haemophilus influenzae* serotype E: microbiological, clinical, and epidemiological features. Clin Infect Dis. 2003;37:841–5. 10.1086/377232 12955648

[28] Talbot B, Alexander E, Lewis S, Newport MJ, Slack MP, Litt DJ, Hepatobiliary infections due to non-capsulated *Haemophilus influenzae.* J Med Microbiol. 2011;60:1383–6. 10.1099/jmm.0.031815-0 21527546

[29] van Wessel K, Rodenburg GD, Veenhoven RH, Spanjaard L, van der Ende A, Sanders EA. Nontypeable *Haemophilus influenzae* invasive disease in The Netherlands: a retrospective surveillance study 2001–2008. Clin Infect Dis. 2011;53:e1–7. 10.1093/cid/cir268 21653293

[30] Farley MM, Stephens DS, Brachman PS Jr, Harvey RC, Smith JD, Wenger JD. Invasive *Haemophilus influenzae* disease in adults. A prospective, population-based surveillance. CDC Meningitis Surveillance Group. Ann Intern Med. 1992;116:806–12. 1314530 10.7326/0003-4819-116-10-806

[31] Bruun B, Gahrn-Hansen B, Westh H, Kilian M. Clonal relationship of recent invasive *Haemophilus influenzae* serotype f isolates from Denmark and the United States. J Med Microbiol. 2004;53:1161–5. 10.1099/jmm.0.45749-0 15496397

[32] Gilsdorf JR. *Haemophilus influenzae* non-type b infections in children. Am J Dis Child. 1987;141:1063–5. 3498361 10.1001/archpedi.141.10.1063

[33] Heath PT, Booy R, Azzopardi HJ, Slack MP, Fogarty J, Moloney AC, Non–type b *Haemophilus influenzae* disease: clinical and epidemiologic characteristics in the *Haemophilus influenzae* type b vaccine era. Pediatr Infect Dis J. 2001;20:300–5. 10.1097/00006454-200103000-00016 11303834

[34] Campos J, Roman F, Perez-Vazquez M, Aracil B, Oteo J, Cercenado E. Antibiotic resistance and clinical significance of *Haemophilus influenzae* type f. J Antimicrob Chemother. 2003;52:961–6. 10.1093/jac/dkh004 14613949

[35] Sarangi J, Cartwright K, Stuart J, Brookes S, Morris R, Slack M. Invasive *Haemophilus influenzae* disease in adults. Epidemiol Infect. 2000;124:441–7. 10.1017/S0950268899003611 10982068 PMC2810930

[36] Cardines R, Giufre M, Mastrantonio P, Ciofi Degli Atti ML, Cerquetti M. Nontypeable *Haemophilus influenzae* meningitis in children: phenotypic and genotypic characterization of isolates. Pediatr Infect Dis J. 2007;26:577–82. 10.1097/INF.0b013e3180616715 17596797

[37] O’Neill JM, St GJ III, Cutter D, Adderson EE, Anyanwu J, Jacobs RF, Invasive disease due to nontypeable *Haemophilus influenzae* among children in Arkansas. J Clin Microbiol. 2003;41:3064–9. 10.1128/JCM.41.7.3064-3069.2003 12843045 PMC165342

[38] Dworkin MS, Park L, Borchardt SM. The changing epidemiology of invasive *Haemophilus influenzae* disease, especially in persons >65 years old. Clin Infect Dis. 2007;44:810–6. 10.1086/511861 17304452

[39] Sill ML, Law DK, Zhou J, Skinner S, Wylie J, Tsang RS. Population genetics and antibiotic susceptibility of invasive *Haemophilus influenzae* in Manitoba, Canada, from 2000 to 2006. FEMS Immunol Med Microbiol. 2007;51:270–6. 10.1111/j.1574-695X.2007.00299.x 17922774

[40] Erwin AL, Sandstedt SA, Bonthuis PJ, Geelhood JL, Nelson KL, Unrath WC, Analysis of genetic relatedness of *Haemophilus influenzae* isolates by multilocus sequence typing. J Bacteriol. 2008;190:1473–83. 10.1128/JB.01207-07 18065541 PMC2238191

