# Supplementary material for: Invasive Haemophilus influenzae Serotype e and f Disease, England and Wales
Source: Emerg Infect Dis. 2012 May;18(5):725–32. doi: 10.3201/eid1805.111738 (PMC3358072; doi:10.3201/eid1805.111738)
Supplement: Related texts

# Research article: Invasive *Haemophilus influenzae* Serotype e and f Disease, England and Wales

**DOI:** 10.3201/eid1805.111738

**Authors:** Shamez N. Ladhani, Sarah Collins, Anna Vickers, David J. Litt, Carina Crawford, Mary E. Ramsay, Mary P.E. Slack

**Affiliations:** Health Protection Agency, London, UK (S.N. Ladhani, S. Collins, A. Vickers, D.J. Litt, C. Crawford, M.E. Ramsay, M.P.E. Slack);; St. George’s University of London, London (S.N. Ladhani)

## Abstract

*Haemophilus influenzae* infection causes serious invasive disease, but incidence of the most virulent serotype, Hib, has dropped since introduction of routine Hib vaccination. In England and Wales, the incidence of 2 other serotypes, Hie and Hif, is increasing; during 2001–2010, there was an 11.0% year-on-year increase in Hif and a 7.4% increase in Hie. In 2009–2010, Hif incidence was 0.090/100,000 persons and Hie incidence 0.030/100,000, with higher rates among infants and older adults. Hie had a more severe clinical course; although outcome at 6 months was comparable for the 2 serotypes, case-fatality rate within 7 days of diagnosis was higher for Hie, even after adjustment for age and comorbidities. Multilocus sequence typing revealed a single major circulating clone for both Hif (sequence type 124; 89/99 isolates, 90%) and Hie (sequence type 18; 21/33, 64%), but no association between type and clinical disease or outcome was found.

*Haemophilus influenzae* is a gram-negative coccobacillus that inhabits the human upper respiratory tract and causes serious invasive infections. The organism can be nonencapsulated (ncHi) or classified into 1 of 6 serotypes (Hia–Hif) on the basis of capsular polysaccharide characteristics. Hib is the most virulent serotype; before routine vaccination was implemented, Hib was responsible for >80% of all invasive *H. influenzae* infections, mainly in children <5 years of age (*1*). Routine immunization with Hib conjugate vaccines, however, has greatly reduced the incidence of invasive Hib disease worldwide (*1*). As a consequence, invasive infections caused by other *H. influenzae* serotypes have become relatively more common.

The United Kingdom introduced the Hib conjugate vaccine in 1992 as a 3-dose infant schedule alongside a 12-month catch-up campaign for all children <5 years of age (*2*). This campaign resulted in a rapid reduction in invasive Hib disease across all age groups (*2*). After 1999, however, Hib disease increased, particularly among toddlers (*3*). Potential explanations for this increase include a greater than expected decline in Hib antibodies after primary immunization, waning of herd immunity after the initial catch-up campaign, and use of a less immunogenic Hib combination vaccine with diphtheria, tetanus, and acellular pertussis (DTaP-Hib) in 2000–2001 (*3*). This resurgence led to establishment of several control measures—re-introduction of a whole-cell pertussis–containing Hib vaccine (DTwP-Hib) in 2002, an Hib booster campaign for toddlers in 2003, and a routine 12-month Hib booster in 2006—that resulted in rapid control of Hib disease (*3*).

Because conjugate vaccines also reduce pharyngeal carriage (*4*), concerns have been raised that other *H. influenzae* strains could fill the ecologic niche and result in invasive disease (serotype replacement) (*5*), although no studies have demonstrated increased carriage rates of other *H. influenzae* serotypes after routine Hib vaccination (*6*). Some countries have reported small but significant increases in invasive ncHi disease after routine Hib vaccination (*7**–**10*); others have not (*11**,**12*). Increases in invasive Hif disease have also been reported, albeit with a small number of cases over years (*8**,**13**–**16*).

In England and Wales, the Health Protection Agency (HPA) has conducted enhanced national *H. influenzae* surveillance (encompassing a denominator population of ≈55 million persons) for >2 decades. The continuing decline in invasive Hib disease meant that in 2009, for the first time, other encapsulated *H. influenzae* serotypes overtook Hib as the most prevalent causes of invasive *H. influenzae* disease (*17*). During 2009–2010, therefore, the HPA investigated the clinical, epidemiologic, and microbiologic characteristics of invasive encapsulated *H. influenzae* disease.

## Methods

The Health and Social Care Act 2001 authorizes the HPA to process confidential patient information for public health purposes (www.legislation.hmso.gov.uk/si/si2002/20021438.htm). HPA provides a national identification and serotyping service for invasive clinical *H. influenzae* isolates for all National Health Service hospital microbiology laboratories in England and Wales through its Haemophilus Reference Unit and conducts enhanced national surveillance through a combination of isolate submission, routine laboratory reporting, and clinical reporting schemes (*3**,**18*). Clinicians, microbiologists, and public health physicians are encouraged to report invasive *H. influenzae* cases to the HPA and submit isolates for species confirmation and serotyping (*19*). The HPA also receives electronic reports of clinically significant invasive pathogens from microbiologists in ≈400 National Health Service laboratories who are routinely contacted to submit isolates to the HPA if not already done, as well as notifications of all *H. influenzae*–related deaths from the Office for National Statistics (ONS) (www.statistics.gov.uk) (*18*).

We defined invasive *H. influenzae* disease as isolation of the organism from a normally sterile site. Localized infections such as pneumonia were included if accompanied by a sterile site isolate. Isolates were confirmed as *H. influenzae* by growth requirement for X and V factors (*20*) and *ompP2*-specific PCR positivity (*21*). Capsulation status was determined by PCR by using *bexA*-specific primers (*22*). Capsular type was confirmed by capsule-specific PCR by using primers for types Hia–Hif (*23*) and slide agglutination (*20*).

Laboratory-confirmed Hif and Hie infections diagnosed during 2009–2010 were followed up 3 months after diagnosis by sending a questionnaire to the patient’s general practitioner (www.hpa.org.uk/Topics/InfectiousDiseases/InfectionsAZ/HaemophilusInfluenzaeTypeB/EnhancedSurveillanceHaemophiliusInfluenzae) and requesting a copy of the hospital discharge summary. A reminder letter was sent to nonresponders after 8 weeks; missing or outstanding information was obtained 1 month later by telephone. Postmortem reports were obtained for all fatal cases that were referred to a coroner. In July 2011, the final outcomes of all patients were confirmed, and causes of death were obtained from the ONS death registrations dataset. Hie/Hif–attributable death was defined as isolation of a pure growth of Hie/Hif from a normally sterile site with clear clinical, radiologic, or histopathologic evidence of invasive bacterial infection and appropriate exclusion of other diagnoses.

Multilocus sequence typing (MLST) was performed according to methods previously described (*24*). Sequence types (STs) were assigned by using an online reference database (http://haemophilus.mlst.net). Minimum-spanning trees were constructed by using Bionumerics version 6.1 software (Advanced Maths, Sint-Martins-Latem, Belgium) as described (*25*).

Data were analyzed by using Stata version 11.0 (StataCorp LP, College Station, TX, USA). Categorical variables were expressed as proportions and compared by using the χ^2^ or Fisher exact test. Data that did not follow a normal distribution were presented as medians with interquartile ranges (IQR). Annual population estimates were obtained from ONS. The binomial method was used to calculate 95% CIs for incidence rates. To estimate any increase in Hie/Hif incidence over time, an overdispersed Poisson regression model was fitted for the annual number of Hie/Hif cases. Yearly changes in population and proportion of total *H. influenzae* isolates serotyped were included in the model as offsets.

## Results

### Epidemiology

During 2001–2010, incidence of invasive Hib disease rose and then declined, while Hif and Hie incidence remained stable (Figure 1). Hif incidence increased 11.0% (95% CI 8.3%–13.8%; p <0.0001) year-on-year, and Hif incidence increased 7.4% (95% CI 0.9%–14.2%; p = 0.024) over the 10 years. Only 6 Hia cases and 2 each of Hic and Hid were reported; none were reported during 2009–1010. In 2009, for the first time, Hif incidence exceeded that of Hib (Figure 1).

**Figure 1:** Epidemiology of invasive encapsulated *Haemophilus influenzae* disease, England and Wales, 2000–2009. Serotypes Hib, Hie, and Hif predominated, with Hib incidence dropping rapidly after re-introduction of a whole-cell pertussis–containing Hib vaccine in 2002, a Hib booster campaign for toddlers in 2003, and a routine 12-month Hib booster in 2006. During the period shown, only 6 cases of Hia and 2 each of Hic and Hid were reported. Error bars indicate 95% CIs.

In 2009–2010, a total of 1,275 invasive *H. influenzae* infections were reported, including 715 (56.1%) ncHi, 69 (5.4%) Hib, 99 (7.8%) Hif, and 33 (2.6%) Hie. In 359 cases (28.2%), the serotype was not known because the isolate was not submitted to the reference laboratory or because the isolate died in transit. Annual incidence of invasive disease was 0.090 (95% CI 0.073–1.10) per 100,000 population for Hif and 0.030 (95% CI 0.021–0.042) per 100,000 for Hie; incidence was highest among infants and older adults (Figure 2).

**Figure 2:** Incidence of invasive *Haemophilus influenzae* serotypes e and f infections, by age group, England and Wales, 2009–2010.

### Clinical Cases During 2009–2010

Clinical questionnaires were completed for all case-patients at a median of 6.8 (IQR 4.4–9.6) months after disease onset. Of the 99 Hif and 33 Hie infections, half (66/132, 50%) occurred in adults >65 years of age (Table). Most case-patients were white (121, 91.7%); 6 were Pakistani, 2 Black African, 2 Indian, and 1 Chinese. Underlying conditions (comorbidities) were present in 105/132 (79.6%) case-patients; these were more prevalent in adults (95/107 [88.8%]) than in children (10/25 [40.0%]; χ^2^ = 29.6; p<0.001) and increased with age: 0/10 (0.0%) infants, 10/15 (66.7%) 1–14-year-olds, 7/10 (70.0%) 15–44-year-olds, 25/31 (79.6%) 45–64-year-olds, and 63/66 (95.5%) >65-year-olds (χ^2^ for trend 41.5; p<0.0001). The prevalence of >2 comorbidities also increased with age, from 0 among infants to 40/66 (60.6%) among >65-year-olds. The overall case-fatality rate (CFR) was 47/132 (35.6%) and also increased with age (1/10 [10.0%], 1/15 [6.7%], 2/10 [20.0%], 8/23 [25.8%] and 35/66 [53.0%], respectively). CFR varied by clinical diagnosis and was highest for pneumonia (36/78, 46.2%) and septicemia (7/18, 38.9%) and lower for other clinical presentations (3/19, 15.9%) and meningitis (1/17, 5.9%)

**Table:** Characteristics of patients with invasive Hie and Hif infections, England and Wales, 2009–2010*

Characteristic	Hie, n = 33	Hif, n = 99	All, n = 132	p value
Median age, y (IQR)	62 (6–79)	65 (46–77)	65 (41–78)	0.54
Male sex	17 (51.5)	47 (47.5)	64 (48.5)	0.69
Underlying conditions				
0	9 (27.3)	18 (18.2)	10 (20.5)	0.26
1	9 (27.3)	32 (32.3)	41 (31.1)	0.59
>2	15 (45.5)	49 (49.5)	64 (48.5)	0.69
Type of underlying condition†				
Heart disease	12 (36.4)	28 (28.3)	40 (30.3)	0.38
Lung disease	7 (21.2)	28 (28.3)	35 (26.5)	0.43
Gastrointestinal disease	4 (12.1)	7 (7.1)	11 (8.3)	0.36
Renal disease	6 (18.2)	12 (12.1)	18 (13.6)	0.38
Metabolic/endocrine	8 (24.2)	13 (13.1)	21 (15.9)	0.13
Malignancy/immunosuppression	4 (12.1)	31 (31.3)	35 (26.5)	0.031
Neurologic condition	3 (9.1)	6 (6.1)	9 (6.8)	0.55
Other risk factors‡	1 (3.0)	5 (5.1)	6 (5.3)	0.63
Type of *Haemophilus influenzae* illness				
Pneumonia	17 (51.5)	61 (61.6)	78 (59.1)	0.47
Meningitis	7 (21.2)	10 (10.1)	17 (12.9)	0.099
Septicemia	2 (6.1)	16 (16.2)	18 (13.6)	0.14
Hepatobiliary infection	3 (9.1)	1 (1.0)	4 (3.0)	0.019
Septic arthritis	0	4 (4.0)	4 (3.0)	0.24
Epiglottitis	1 (3.0)	3 (3.0)	4 (3.0)	1.00
Cellulitis	1 (3.0)	4 (4.0)	5 (3.8)	0.79
Urogenital infection	1 (3.0)	0	1 (0.8)	0.082
Endocarditis	1 (3.0)	0	1 (0.8)	0.082
CFR				
Overall	10/33 (41.7)	34/99 (42.0)	44/132 (41.9)	0.98
No underlying condition	3/9 (33.3)	0/18	3/27 (11.1)	0.009
1 underlying condition	5/24 (20.8)	9/81 (11.1)	14/105 (13.3)	0.22
>2 underlying conditions	3/15 (20.0)	7/49 (14.3)	10/64 (15.6)	0.59
Attributable CFR	10/33 (30.3)	12/99 (12.1)	22/132 (16.7)	0.015
CFR within 7 d	8/33 (24.2)	8/99 (8.8)	16/132 (12.1)	0.014
CFR within 28 d	10/33 (30.3)	15/99 (15.2)	25/132 (18.9)	0.054
CFR for meningitis§	1/7 (14.3)	0/10	1/17 (5.9)	0.22
CFR for pneumonia§	7/17 (41.2)	7/61 (11.5)	14/78 (18.0)	0.005
CFR for septicemia§	0/2	1/16 (6.3)	1/18 (5.6)	0.72
CFR for other conditions§	0/7	0/12	0/19	

*Values are no. cases (%) or no. cases/no. in category (%), unless otherwise specified. Hie, *Haemophilus influenzae* serotype e; Hif, *H. influenzae* serotype f; IQR, interquartile range; CFR, case-fatality ratio. †Some case-patients had >1 underlying condition. ‡For Hie, long-term complications of extreme prematurity (n = 1); for Hif, alcohol dependency (n = 3), long-term complications of extreme prematurity (n = 1), and schizophrenia (n = 1). §Death within 7 d.

Patients with Hif and Hie infections did not differ significantly by sex, age distribution, or presence or number of comorbidities, although malignancy/immunosuppression was overrepresented among Hif infections (Table). In particular, 27/99 (27.3%) patients with Hif infection had malignancy: 10 (37.0%) with solid organ tumors, 9 (33.3%) with myeloma, and 8 (29.6%) with other hematologic malignancies. By comparison, 1 Hie patient had a solid organ tumor (1/33, 3.0%; χ^2^ = 8.7; p = 0.003). In 1 patient, Hif infection was the initial indication of myeloma. Initial signs and symptoms for Hif versus Hie were also similar, except for hepatobiliary infection, which was more prevalent among Hie case-patients (Table). Also, meningitis was ≈2× more common among patients with Hie, although this finding was not statistically significant. Among case-patients with meningitis, 5/7 (71.4%) Hie were previously healthy; 1/7 (14.3%) died, and half the survivors (3/6) had long-term sequelae. In contrast, only 3/10 (30.0%) Hif meningitis patients were previously healthy; all recovered without complications.

CFRs were similar for patients with Hif and Hie overall and for those with comorbidities but significantly lower for previously healthy patients with Hif versus Hie (0/18 [0.0%] vs. 3/9 [33.3%]; χ^2^ = 6.75; p = 0.009). Among Hif case-patients, CFR increased with the number of comorbidities: 0 for no comorbidities, 11/32 (34.4%) for 1 comorbidity, and 23/49 (46.9%) for >2 comorbidities (χ^2^ = 11.7; p = 0.002). This trend was not observed for Hie case-patients (3/9 [33.3%], 4/9 [55.5%], and 5/15 [33.3%], respectively). After adjustment for age and comorbidity, patients who had Hie had a 2.6× greater risk for death than did those who had Hif (hazard ratio 2.63, 95% CI 1.14–6.10; p = 0.024). Moreover, the interval between diagnosis and death was significantly shorter for Hie (Table), even after adjustment for age and comorbidities (Figure 3). Among case-patients with pneumonia, CFR within 7 days after infection was higher for those with Hie than Hif (Table); this difference remained significant even after adjusting for age and comorbidity (OR 5.1, 95% CI 1.4–18.6; p = 0.014).

**Figure 3:** Kaplan-Meier curve for outcome among patients with *Haemophilus influenzae* serotype e and f infections, after adjustment for age and comorbidities, England and Wales, 2009–2010.

### Infections in Children

Children <15 years of age accounted for 25/132(18.9%) Hie/Hif infections. Ten cases (7 Hif, 3 Hie) occurred in the first year of life, with patient age evenly distributed from 1 to 10 months of age and no cases among neonates. None of the infants had been born prematurely or had comorbidities; 9/10 had meningitis, and 1 had Hif septic arthritis. All Hif case-patients recovered uneventfully, but all 3 Hie case-patients had meningitis and either died of purulent meningitis (1 patient) or had long-term sequelae (bilateral sensorineural deafness [1] or seizures [1]).

Among children 1–4 years of age, 8 had Hif and 5 had Hie infection. Of the 8 Hif case-patients, 6 had comorbidities: malignancy/immunosuppression (4 patients) or chronic lung disease of prematurity (2; pneumonia developed in both). There were 2 other cases of pneumonia, 1 of meningitis in a previously healthy child, 2 of septicemia, and 1 of cellulitis; none of the patients needed intensive care, and all survived. Two of the Hie case-patients had comorbidities, 1 with multiple complications of extreme prematurity who developed meningitis and 1 with complex congenital heart disease who developed endocarditis. Three previously healthy toddlers developed cellulitis (recovered without complications), meningitis (bilateral sensorineural deafness developed), and pneumonia (died).

Among older children 5–14 years of age, only 2 had Hie/Hif infections: 1 Hif septicemia case in a child with malignancy, and 1 Hie meningitis case after otitis media in a previously healthy child. Both patients recovered uneventfully.

### Adults 15–64 Years of Age

Among 15–44-year-olds, 8 Hif and 2 Hie infections occurred. Comorbidities were present in 5/8 Hif case-patients. Pneumonia was the most common diagnosis (4 patients), followed by septicemia (3) and septic arthritis (1). Two Hif septicemia cases occurred in pregnant women at 8 and 25 weeks’ gestation; 1 infection resulted in septic abortion. One man who had a comorbid malignancy died within 7 days after infection. Both Hie case-patients had comorbidities: 1 had chronic liver disease, had septicemia develop, and died >1 month later of the underlying disease; 1 had HIV, had pneumonia develop, and survived.

Among 45–64-year-olds, 23 had Hif and 8 had Hie infection. Most Hif cases had comorbidities (20/23, 87.0%) and an initial diagnosis of pneumonia (14/23, 60.9%); 3 cases each had an initial diagnosis of meningitis and septicemia. Of the 6/23 (26.1%) case-patients who died, 1 with chronic obstructive pulmonary disease died of pneumonia within 7 days, and 5 died of an underlying malignancy >3 months later. The 3 previously healthy Hif case-patients had pneumonia (2 patients) and septic arthritis (1) and recovered uneventfully. Hie case-patients had pneumonia (5), meningitis (1), cholangitis (1), and cholecystitis (1); 6/8 had comorbidities. Two Hie case-patients with comorbidities died, 1 within 7 days after multiorgan failure, and 1 after 3 months of underlying malignancy.

### Adults >65 Years of Age

Half the infections (52 Hif, 14 Hie) occurred in this age group. Fifty (96.2%) Hif case-patients had comorbidities (11 with malignancy), and most had pneumonia (35 patients) or septicemia (9). CFR was highest in this age group (27/50, 54.0%), although only 6 (12.0%) case-patients died within 7 days and an additional 13 (26.0%) within 28 days. The 2 previously healthy Hif case-patients had initial diagnoses of pneumonia and supraglottitis but recovered uneventfully. Of the 14 Hie infections, 13 had comorbidities (only 2 had malignancy). Pneumonia (10 patient) was the most common clinical presentation; 1 case each of supraglottitis, urogenital septicemia, cholangitis, and cholecystitis occurred. Eight of 14 (57.1%) Hie case-patients died; 6 with pneumonia and 1 with biliary sepsis died within 7 days, and 1 died later of underlying malignancy.

### Characterization of Hie and Hif Isolates

Characterization of clinical isolates by MLST showed 2 distinct phylogenetic clusters around a dominant ST within each serotype (Figure 4). Twenty-one (64%) Hie isolates were ST18, while other isolates diverged from this ST at 1 (single-locus variant) or 2 (double-locus variant) of 7 genomic MLST loci. Among Hif isolates, 89 (90%) were ST124; 4 STs were single- or double-locus variants of this ST, and the remaining 2 differed from ST124 at 3 (ST968) and 4 (ST16) loci. STs 966–970 and 973 are previously unreported types assigned as a result of this study. Except for ST16 (which shared its *atpG* allele with all Hie isolates) and ST66 (which shared its *fucK* allele with most Hif isolates), the 2 serotype clusters did not share any individual MLST alleles in common. Similarly, apart from occasional shared *fucK* alleles, neither cluster had alleles in common with contemporary Hib isolates (data not shown). A minimum-evolution tree constructed from concatenated DNA sequences from the 7 MLST loci suggested that the Hie and Hif isolates in this study were evolutionarily closely related to each other but distantly related to 64 Hib isolates from the same period (data not shown).

**Figure 4:** Minimum spanning tree showing the relationship between multilocus sequence types of 33 *Haemophilus influenzae* serotype e (Hie, red) and 99 Hif (green) strains isolated from patients in England and Wales, 2009–2010. The tree was derived from the 7 multilocus sequence type alleles of each isolate; the number within each circle represents a unique sequence type, and the size of the circle illustrates the proportion of strains with that sequence type (the smallest circle represents 1 isolate). Thick lines, thin lines, and dotted lines separating circles indicate single-, double-, and triple-locus variants, respectively.

## Discussion

In England and Wales, encapsulated *H. influenzae* other than Hib rarely cause invasive disease; among those, Hie and Hif are most common. These serotypes are similar to ncHi in that they cause invasive disease mainly in older adults, who often have comorbidities and present with pneumonia. However, although Hie is 3× less common than Hif, it appears to be more virulent, with more complications of meningitis and higher infection-attributable CFR, even after adjustment for age and comorbidities.

The HPA national reference service for *H. influenzae* species confirmation and serotyping provides a rich source of epidemiologic and molecular surveillance data, and the consistently high proportion of serotyped isolates with data enhancement from multiple sources further strengthens the quality and completeness of the surveillance program (*18*). This data source enables more accurate interpretation of trends in disease rates and pathogens over time, as demonstrated by the relatively stable trends in the rare *H. influenzae* serotypes over the past decade. Despite the small number of cases, our results show small but gradual annual increases in invasive Hie and Hif disease. These increases are most likely to be a consequence of 1) the expansion of at-risk populations in industrialized countries resulting from increased survival rates among those at the extremes of age, who often have long-term comorbidities; 2) better survival prognosis among patients with malignancy; and 3) increased use of immunosuppressive therapy for immunologic and rheumatologic conditions.

Other countries have also reported an increase in invasive Hif disease since routine Hib vaccination was introduced. In 1 surveillance study involving multiple states in the United States, Hif incidence increased from 0.14/100,000 population in 1989 to 1.9/100,000 in 1994 (*13*); in Utah, Hif incidence increased from 0.14/100,000 person-years during 1998–2008 to 0.48/100,000 during 2007–2008 (*8*). The US Active Bacterial Core surveillance also recently reported an increase in invasive Hif disease, from 0.06 cases/100,000 population in 1989 to 0.25/100,000 in 2008 (*16*). In Sweden, invasive Hif disease increased 2.3% annually during 1997–2009 (*10*); however, other studies, including a recent study in Europe involving national surveillance data from 14 countries over 11 years, did not show any changes in Hif incidence (*7**,**26*). In relation to Hie, perhaps because of the small number of invasive cases caused by this serotype, we identified only the recent US Active Bacterial Core surveillance study that reported a small increase in disease incidence over 2 decades (*16*).

In addition to national epidemiologic surveillance, we prospectively collected clinical information for all laboratory-confirmed infections. Most published reports of Hie and Hif infection are in the form of individual cases, with few studies reporting a sufficient number of cases focusing on clinical aspects of specific *H. influenzae* serotypes (*10**,**13**,**27*). Our results indicate that Hie and Hif share many features characteristic of invasive ncHi disease, such as patient age distribution, higher risk for infection among vulnerable populations, and higher CFR compared with Hib (*7*). Certain features that were previously attributed primarily to ncHi, such as causing hepatobiliary disease (*28**,**29*) and disease during pregnancy (*30*), were also observed among Hie and Hif case-patients, respectively, in our study. The proportion of case-patients with comorbidities in our cohort was similar to previous smaller studies (60%–80%) (*13**,**27**,**31**–**34*) and to that among ncHi case-patients (*13**,**26**,**33**,**35**–**37*). Several Hif case-patients had disorders of B-cell immunity, such as chronic lymphocytic leukemia and multiple myeloma, which have also been reported among persons with invasive ncHi; this finding suggests a major role for humoral immunity (*10*). Declining B-cell function has also been speculated to explain the increasing incidence with age (*10*). The finding that none of the infants in this study had any reported comorbidities at infection, however, is intriguing. Most children and adults outside this age group had comorbidities, which suggests that some of these infants might have as-yet-undetected subtle immunologic abnormalities that made them vulnerable to opportunistic infections.

Although Hie and Hif should be compared with caution because of the relatively small number of cases, our results suggest that Hie causes more severe clinical disease. This observation is supported by recent population-based studies that have reported a worse outcome for patients with Hie than for those with Hif, including a CFR of 24% versus 10.0% (p = 0.003) in a study in Europe (*7*) and 32% versus 6% (p = 0.002) in a US study (*38*). One possible reason that Hie has a lower incidence but it is more fatal than Hif could be that this serotype is less pathogenic and infects persons who are older and/or in much poorer health and who, therefore, are more likely to die. This hypothesis, however, is not supported by our study because the median age at disease onset and the prevalence of any or multiple comorbidities were similar, and the 7-day CFR remained significant even after adjusting for these risk factors. However, the 6-month CFR for the 2 serotypes was similar, which suggests that these pathogens can infect highly vulnerable persons who, even if they survived the infection, subsequently succumbed to their underlying illness; this finding has been also observed for invasive ncHi disease (*10**,**35*). For example, a recent study in Sweden reported that the 28-day CFR for invasive ncHi disease was 8%, but 1-year CFR increased to 29% (*10*).

MLST analysis of the clinical isolates revealed dominant Hie (ST18) and Hif (ST124) types that have been reported among bacterial strains from Europe and North America (http://haemophilus.mlst.net) (*24*). In 2 recent independent studies in Canada, ST124 was also the dominant Hif type, and the Hie types detected differed from ST18 at only a single MLST locus (*9**,**39*). None of the Hie or Hif MLST types in our study have been reported outside of their respective serotypes. These observations, combined with phylogenetic analysis of MLST sequences across capsulated and noncapsulated *H. influenzae* isolates in our and other studies (*24**,**40*), suggest that any increase in Hie and Hif disease is not due to genetic capsule switching events between these serotypes and Hib.

In conclusion, invasive Hie and Hif infections are rare in England and Wales, but their incidence is increasing slowly. The clinical features are similar to ncHi, although Hie appears to be associated with more severe disease and worse infection-attributable outcome. Given that most infections occur among persons with comorbidities, it would be prudent to investigate all previously healthy persons in whom Hie/Hif infections develop for possible underlying immune deficiency, malignancy, or other undiagnosed conditions. Phylogenetic analysis of clinical isolates showed no evidence of serotype replacement by capsule switching between Hie or Hif and Hib and no association between sequence types and clinical disease or outcome.

## References

[1] Peltola H. Worldwide *Haemophilus influenzae* type b disease at the beginning of the 21st century: global analysis of the disease burden 25 years after the use of the polysaccharide vaccine and a decade after the advent of conjugates. Clin Microbiol Rev. 2000;13:302–17. 10.1128/CMR.13.2.302-317.200010756001PMC100154

[2] Heath PT, Booy R, Azzopardi HJ, Slack MP, Bowen-Morris J, Griffiths H, Antibody concentration and clinical protection after Hib conjugate vaccination in the United Kingdom. JAMA. 2000;284:2334–40. 10.1001/jama.284.18.233411066183

[3] Ladhani S, Slack MP, Heys M, White J, Ramsay ME. Fall in *Haemophilus influenzae* serotype b (Hib) disease following implementation of a booster campaign. Arch Dis Child. 2008;93:665–9. 10.1136/adc.2007.12688817942585

[4] Takala AK, Eskola J, Leinonen M, Kayhty H, Nissinen A, Pekkanen E, Reduction of oropharyngeal carriage of *Haemophilus influenzae* type b (Hib) in children immunized with an Hib conjugate vaccine. J Infect Dis. 1991;164:982–6. 10.1093/infdis/164.5.9821940479

[5] Tsang R. Capsule switching and capsule replacement in vaccine-preventable bacterial diseases. Lancet Infect Dis. 2007;7:569–70. 10.1016/S1473-3099(07)70191-317714669

[6] Lowther SA, Shinoda N, Juni BA, Theodore MJ, Wang X, Jawahir SL, *Haemophilus influenzae* type b infection, vaccination, and H. influenzae carriage in children in Minnesota, 2008–2009. Epidemiol Infect. 2012;140:566–74. 10.1017/S095026881100079321676359

[7] Ladhani S, Slack MP, Heath PT, von Gottberg A, Chandra M, Ramsay ME. European Union Invasive Bacterial Infection Surveillance participants. Invasive *Haemophilus influenzae* disease, Europe, 1996–2006. Emerg Infect Dis. 2010;16:455–63.2020242110.3201/eid1603.090290PMC3322004

[8] Rubach MP, Bender JM, Mottice S, Hanson K, Weng HY, Korgenski K, Increasing incidence of invasive *Haemophilus influenzae* disease in adults, Utah, USA. Emerg Infect Dis. 2011;17:1645–50. 10.3201/eid1709.10199121888789PMC3322072

[9] Shuel M, Hoang L, Law DK, Tsang R. Invasive *Haemophilus influenzae* in British Columbia: non-Hib and non-typeable strains causing disease in children and adults. Int J Infect Dis. 2011;15:e167–73. 10.1016/j.ijid.2010.10.00521134777

[10] Resman F, Ristovski M, Ahl J, Forsgren A, Gilsdorf JR, Jasir A, Invasive disease caused by *Haemophilus influenzae* in Sweden 1997–2009; evidence of increasing incidence and clinical burden of non–type b strains. Clin Microbiol Infect. 2011;17:1638–45. 10.1111/j.1469-0691.2010.03417.x21054663

[11] Kalies H, Siedler A, Grondahl B, Grote V, Milde-Busch A. von KR. Invasive *Haemophilus influenzae* infections in Germany: impact of non-type b serotypes in the post-vaccine era. BMC Infect Dis. 2009;9:45. 10.1186/1471-2334-9-4519379490PMC2678273

[12] Giufrè M, Cardines R, Caporali MG, Accogli M, D’Ancona F, Cerquetti M. Ten years of Hib vaccination in Italy: prevalence of non-encapsulated *Haemophilus influenzae* among invasive isolates and the possible impact on antibiotic resistance. Vaccine. 2011;29:3857–62. 10.1016/j.vaccine.2011.03.05921459175

[13] Urwin G, Krohn JA, Deaver-Robinson K, Wenger JD, Farley MM. Invasive disease due to *Haemophilus influenzae* serotype f: clinical and epidemiologic characteristics in the H. influenzae serotype b vaccine era. The *Haemophilus influenzae* Study Group. Clin Infect Dis. 1996;22:1069–76. 10.1093/clinids/22.6.10698783712

[14] Resman F, Svensj T, Nal C, Cronqvist J, Brorson HK, Odenholt I, Necrotizing myositis and septic shock caused by *Haemophilus influenzae* type f in a previously healthy man diagnosed with an IgG3 and a mannose-binding lectin deficiency. Scand J Infect Dis. 2011;43:972–6. 10.3109/00365548.2011.58907921728743

[15] Adam HJ, Richardson SE, Jamieson FB, Rawte P, Low DE, Fisman DN. Changing epidemiology of invasive *Haemophilus influenzae* in Ontario, Canada: evidence for herd effects and strain replacement due to Hib vaccination. Vaccine. 2010;28:4073–8. 10.1016/j.vaccine.2010.03.07520398617

[16] MacNeil JR, Cohn AC, Farley M, Mair R, Baumbach J, Bennett N, Current epidemiology and trends in invasive *Haemophilus influenzae* disease—United States, 1989–2008. Clin Infect Dis. 2011;53:1230–6. 10.1093/cid/cir73522080119

[17] Laboratory reports of *Haemophilus influenzae* by age group and serotype, England and Wales, fourth quarter, 2010 (and 2009). Health Protection Reports. 2011;5:6–7.

[18] Ladhani S, Slack MP, Heath PT, Ramsay ME. Changes in ascertainment of Hib and its influence on the estimation of disease incidence in the United Kingdom. Epidemiol Infect. 2007;135:861–7. 10.1017/S095026880600738217092395PMC2870625

[19] Heath PT, Booy R, Griffiths H, Clutterbuck E, Azzopardi HJ, Slack MP, Clinical and immunological risk factors associated with *Haemophilus influenzae* type b conjugate vaccine failure in childhood. Clin Infect Dis. 2000;31:973–80. 10.1086/31813211049779

[20] Slack MPE. *Haemophilus*. In: Borriello SP, Murray PR, Funke G, editors. Topley and Wilson’s microbiology and microbial infections, vol. 2. London: Hodder Arnold Ltd.; 1995. p. 1692–718.

[21] Hobson RP, Williams A, Rawal K, Pennington TH, Forbes KJ. Incidence and spread of *Haemophilus influenzae* on an Antarctic base determined using the polymerase chain reaction. Epidemiol Infect. 1995;114:93–103. 10.1017/S09502688000519437867747PMC2271340

[22] van Ketel RJ. de WB, van AL. Detection of *Haemophilus influenzae* in cerebrospinal fluids by polymerase chain reaction DNA amplification. J Med Microbiol. 1990;33:271–6. 10.1099/00222615-33-4-2712258914

[23] Falla TJ, Crook DW, Brophy LN, Maskell D, Kroll JS, Moxon ER. PCR for capsular typing of *Haemophilus influenzae.* J Clin Microbiol. 1994;32:2382–6.781447010.1128/jcm.32.10.2382-2386.1994PMC264070

[24] Meats E, Feil EJ, Stringer S, Cody AJ, Goldstein R, Kroll JS, Characterization of encapsulated and noncapsulated *Haemophilus influenzae* and determination of phylogenetic relationships by multilocus sequence typing. J Clin Microbiol. 2003;41:1623–36. 10.1128/JCM.41.4.1623-1636.200312682154PMC153921

[25] Litt DJ, Neal SE, Fry NK. Changes in genetic diversity of the *Bordetella pertussis* population in the United Kingdom between 1920 and 2006 reflect vaccination coverage and emergence of a single dominant clonal type. J Clin Microbiol. 2009;47:680–8. 10.1128/JCM.01838-0819158267PMC2650949

[26] Campos J, Hernando M, Roman F, Perez-Vazquez M, Aracil B, Oteo J, Analysis of invasive *Haemophilus influenzae* infections after extensive vaccination against H. influenzae type b. J Clin Microbiol. 2004;42:524–9. 10.1128/JCM.42.2.524-529.200414766811PMC344522

[27] Campos J, Roman F, Perez-Vazquez M, Oteo J, Aracil B, Cercenado E. Infections due to *Haemophilus influenzae* serotype E: microbiological, clinical, and epidemiological features. Clin Infect Dis. 2003;37:841–5. 10.1086/37723212955648

[28] Talbot B, Alexander E, Lewis S, Newport MJ, Slack MP, Litt DJ, Hepatobiliary infections due to non-capsulated *Haemophilus influenzae.* J Med Microbiol. 2011;60:1383–6. 10.1099/jmm.0.031815-021527546

[29] van Wessel K, Rodenburg GD, Veenhoven RH, Spanjaard L, van der Ende A, Sanders EA. Nontypeable *Haemophilus influenzae* invasive disease in The Netherlands: a retrospective surveillance study 2001–2008. Clin Infect Dis. 2011;53:e1–7. 10.1093/cid/cir26821653293

[30] Farley MM, Stephens DS, Brachman PS Jr, Harvey RC, Smith JD, Wenger JD. Invasive *Haemophilus influenzae* disease in adults. A prospective, population-based surveillance. CDC Meningitis Surveillance Group. Ann Intern Med. 1992;116:806–12.131453010.7326/0003-4819-116-10-806

[31] Bruun B, Gahrn-Hansen B, Westh H, Kilian M. Clonal relationship of recent invasive *Haemophilus influenzae* serotype f isolates from Denmark and the United States. J Med Microbiol. 2004;53:1161–5. 10.1099/jmm.0.45749-015496397

[32] Gilsdorf JR. *Haemophilus influenzae* non-type b infections in children. Am J Dis Child. 1987;141:1063–5.349836110.1001/archpedi.141.10.1063

[33] Heath PT, Booy R, Azzopardi HJ, Slack MP, Fogarty J, Moloney AC, Non–type b *Haemophilus influenzae* disease: clinical and epidemiologic characteristics in the *Haemophilus influenzae* type b vaccine era. Pediatr Infect Dis J. 2001;20:300–5. 10.1097/00006454-200103000-0001611303834

[34] Campos J, Roman F, Perez-Vazquez M, Aracil B, Oteo J, Cercenado E. Antibiotic resistance and clinical significance of *Haemophilus influenzae* type f. J Antimicrob Chemother. 2003;52:961–6. 10.1093/jac/dkh00414613949

[35] Sarangi J, Cartwright K, Stuart J, Brookes S, Morris R, Slack M. Invasive *Haemophilus influenzae* disease in adults. Epidemiol Infect. 2000;124:441–7. 10.1017/S095026889900361110982068PMC2810930

[36] Cardines R, Giufre M, Mastrantonio P, Ciofi Degli Atti ML, Cerquetti M. Nontypeable *Haemophilus influenzae* meningitis in children: phenotypic and genotypic characterization of isolates. Pediatr Infect Dis J. 2007;26:577–82. 10.1097/INF.0b013e318061671517596797

[37] O’Neill JM, St GJ III, Cutter D, Adderson EE, Anyanwu J, Jacobs RF, Invasive disease due to nontypeable *Haemophilus influenzae* among children in Arkansas. J Clin Microbiol. 2003;41:3064–9. 10.1128/JCM.41.7.3064-3069.200312843045PMC165342

[38] Dworkin MS, Park L, Borchardt SM. The changing epidemiology of invasive *Haemophilus influenzae* disease, especially in persons >65 years old. Clin Infect Dis. 2007;44:810–6. 10.1086/51186117304452

[39] Sill ML, Law DK, Zhou J, Skinner S, Wylie J, Tsang RS. Population genetics and antibiotic susceptibility of invasive *Haemophilus influenzae* in Manitoba, Canada, from 2000 to 2006. FEMS Immunol Med Microbiol. 2007;51:270–6. 10.1111/j.1574-695X.2007.00299.x17922774

[40] Erwin AL, Sandstedt SA, Bonthuis PJ, Geelhood JL, Nelson KL, Unrath WC, Analysis of genetic relatedness of *Haemophilus influenzae* isolates by multilocus sequence typing. J Bacteriol. 2008;190:1473–83. 10.1128/JB.01207-0718065541PMC2238191

# Research article: Invasive *Haemophilus influenzae* Serotype e and f Disease, England and Wales

**DOI:** 10.3201/eid1805.111738

**Authors:** Shamez N. Ladhani, Sarah Collins, Anna Vickers, David J. Litt, Carina Crawford, Mary E. Ramsay, Mary P.E. Slack

**Affiliations:** Health Protection Agency, London, UK (S.N. Ladhani, S. Collins, A. Vickers, D.J. Litt, C. Crawford, M.E. Ramsay, M.P.E. Slack);; St. George’s University of London, London (S.N. Ladhani)

## Medscape CME Quiz

## Earning CME Credit

To obtain credit, you should first read the journal article. After reading the article, you should be able to answer the following, related, multiple-choice questions. To complete the questions (with a minimum 70% passing score) and earn continuing medical education (CME) credit, please go to www.medscape.org/journal/eid. Credit cannot be obtained for tests completed on paper, although you may use the worksheet below to keep a record of your answers. You must be a registered user on Medscape.org. If you are not registered on Medscape.org, please click on the New Users: Free Registration link on the left hand side of the website to register. Only one answer is correct for each question. Once you successfully answer all post-test questions you will be able to view and/or print your certificate. For questions regarding the content of this activity, contact the accredited provider, CME@medscape.net. For technical assistance, contact CME@webmd.net. American Medical Association’s Physician’s Recognition Award (AMA PRA) credits are accepted in the US as evidence of participation in CME activities. For further information on this award, please refer to http://www.ama-assn.org/ama/pub/category/2922.html. The AMA has determined that physicians not licensed in the US who participate in this CME activity are eligible for *AMA PRA Category 1 Credits*™. Through agreements that the AMA has made with agencies in some countries, AMA PRA credit may be acceptable as evidence of participation in CME activities. If you are not licensed in the US, please complete the questions online, print the certificate and present it to your national medical association for review.

## Article Title: Invasive *Haemophilus influenzae* Serotype e and f Disease, England and Wales

## CME Questions

**1. Which of the following statements regarding the overall epidemiology of *Haemophilus influenzae* f (Hif) and *Haemophilus influenzae* e (Hie) in the current study is most accurate?**

A. There was a progressive increase in the incidence of Hif

B. Rates of Hie rose between 2001 and 2006, and then fell significantly

C. The annual incidence of invasive Hie disease was higher than the rate of invasive Hif disease

D. Older adults were much more likely to have Hif vs Hie infection

**2. Which of the following statements regarding mortality related to Hif and Hie in the current study is most accurate?**

A. The overall case fatality ratio was higher for Hif vs Hie

B. After adjustment, the risk of dying was higher with Hie vs Hif

C. Hif pneumonia was particularly associated with a higher risk of mortality compared with Hie pneumonia

D. Most fatal cases of invasive Hif and Hie were due to septicemia

**3. What should you consider regarding infection with Hif and Hie among children and adolescents in the current study?**

A. Children accounted for more than half of all cases

B. Nearly all infections among children less than one year old were clustered in the age range of less than 3 months of age

C. Most cases among infants involved meningitis

D. Cases were evenly distributed between early and late childhood

**4. What should you consider regarding infection with Hif and Hie among older adults in the current study?**

A. Older adults accounted for half of all cases

B. Only 30% of cases had comorbidity

C. Septicemia was the most common presentation of illness

D. They demonstrated infection with a wide variety of phylogenetic types of Hif and Hie

## Activity Evaluation

**Table:** 

1. The activity supported the learning objectives.				
Strongly Disagree				Strongly Agree
1	2	3	4	5
2. The material was organized clearly for learning to occur.				
Strongly Disagree				Strongly Agree
1	2	3	4	5
3. The content learned from this activity will impact my practice.				
Strongly Disagree				Strongly Agree
1	2	3	4	5
4. The activity was presented objectively and free of commercial bias.				
Strongly Disagree				Strongly Agree
1	2	3	4	5
